# Epilepsy in Children with Autistic Spectrum Disorder

**DOI:** 10.3390/children6020015

**Published:** 2019-01-25

**Authors:** Iliyana Pacheva, Ivan Ivanov, Ralitsa Yordanova, Katerina Gaberova, Fani Galabova, Margarita Panova, Aneliya Petkova, Elena Timova, Iglika Sotkova

**Affiliations:** 1 Department of Pediatrics and Medical Genetics, Medical University, 4000 Plovdiv, Bulgaria; ivanovist@gmail.com (I.I.); ralitsa_iordanova@yahoo.com (R.Y.); panova_marg@mail.bg (M.P.); iglika26@abv.bg (I.S.); 2 Department of Pediatrics, University Hospital “St. George”, 4000 Plovdiv, Bulgaria; katerina_gaberowa@yahoo.com (K.G.); fanita_g@abv.bg (F.G.); anelia75@mail.bg (A.P.); etimova@gmail.com (E.T.)

**Keywords:** epilepsy, autism, ASD, intellectual disability, children, EEG

## Abstract

The comorbidity of autistic spectrum disorder (ASD) and epilepsy has been widely discussed but many questions still remain unanswered. The aim of this study was to establish the occurrence of epilepsy among children with ASD to define the type of epileptic seizures and syndromes, the age of onset of epilepsy, EEG abnormalities, the used antiepileptic drugs and the therapeutic responses for seizures and autistic behavior, as well as to find some correlations between epilepsy and gender, etiology and intellectual disability (ID). A retrospective study of medical files of 59 patients (aged 1–18 years) with ASD during a 5-year period was performed. ASD diagnosis was based on the DSM-5 diagnostic criteria. The patients were examined with a detailed medical history, physical and neurological examination, as well as some additional functional, imaging, laboratory and genetic investigations ASD etiology was syndromic in 9, probable syndromic in 9, and idiopathic in 41 children. ID was established in 90% of ASD children, and epilepsy in 44.4%. The onset of epilepsy prevailed before 7 years of age. The most common seizure types were focal with or without secondary generalization (53.4%). Focal epileptiform EEG abnormalities prevailed. Therapeutic response to seizures was good: 58% were seizure-free, while 27% had >50% seizure reduction but no improvement in autistic behavior. There was no correlation between epilepsy and either occurrence or degree of ID. There was a correlation between the frequency of epileptic seizures and the degree of ID. There was no significant difference among epilepsy rates in different etiologic, gender, and ID groups, probably because of the high percentage of ID and because this was a hospital-based study. Our study showed a significant percentage of epilepsy in ASD population and more than 1/4 were of symptomatic etiology. Those could be managed with specific treatments based on the pathophysiology of the gene defect.

## 1. Introduction

According to the literature data, epilepsy is more common among individuals with autistic spectrum disorder (ASD) (5–46%) compared to the general population (0.5–1%) [1,2,3,4,5,6,7,8,9].

Frequently asked questions about autism and epilepsy are:

- Is there a real association between epilepsy and autism?
- If we suggest a link between epilepsy and autism, will this contribute to solving the etiology of autism?
- Does a particular therapeutic approach to the epilepsy of autistic patients exist and is it the same for all autistic children?
- Could achieving therapeutic control of the epilepsy, i.e., making patients seizure-free, improve autistic behavior and/or intellectual disability (ID)?
- Does the treatment of epileptiform EEG abnormalities improve autistic behavior and cognitive functions, and should epileptiform EEG abnormalities be treated?

It is interesting what we have learned about autism since Kanner’s time up to now: Autism is not a single disorder but a spectrum of closely related disorders with shared core symptoms, so ASD is now considered the proper term [10]. ASD is a group of developmental disorders which begin in early childhood and can be diagnosed even before 3 years of age [11]. ASD is not so rare, as its incidence is about 1 per 100 individuals [12]. ASD has underlying neurobiological mechanisms [13,14,15]. There are many comorbid conditions that could worsen the life of autistic patients [16].

There is much evidence in the literature that the etiology of autism is complex [15,17,18]. A number of genetic, epigenetic and environmental factors influence neuronal networks, cortical-subcortical connectivity, GABAergic interneurons, neurotransmitters and their function through molecular and cellular pathophysiology and thus play a role for the autistic phenotype [13,19,20,21,22]. ASD can be considered symptomatic–syndromic (i.e., associated with dysmorphic signs, cutaneous lesions, microcephaly, macrocephaly, neuromuscular disease or other distinctive clinical features, metabolic diseases or structural brain abnormalities) or idiopathic–non-syndromic without any symptoms suggesting a certain etiology [18,21,23]. Electrical status epilepticus in slow wave sleep (ESES), a clinical entity associated with the regression of language and behavioral changes such as ASD-associated regression could also be related to syndromic autism when it occurs in early life [24].

The comorbidity of autism and epilepsy has been widely discussed in the literature; however, independent of many possible explanations, the association between these two conditions has still remained obscure and additional research and thorough analyses are needed to decode the possible mechanisms of this link [3,7,19,20,22].

The aim of this study was to establish the occurrence of epilepsy among children with ASD, to define the type of epileptic seizures and syndromes, the age of onset of epilepsy, the frequency of epileptic seizures, the used antiepileptic drugs and the therapeutic responses for seizures and autistic behavior and EEG abnormalities, as well as to find some correlations between epilepsy and other factors, such as the etiology of ASD and intellectual disability.

## 2. Patients and Methods

A retrospective study of medical files of 59 patients up to 18 years of age (39 males and 20 females, mean age 7 years7 months) admitted to the Pediatric Department of St. George University Hospital in Plovdiv with a diagnosis of ASD during a 5-year period (January 2014–June 2018) was performed. ASD diagnosis was based on the DSM-5 diagnostic criteria obtained by two neurologists, a psychiatrist and two psychologists [10].

The patients were examined with a detailed medical history, physical and neurological examination, as well as some additional functional, imaging, laboratory and genetic investigations such as CT, MRI, biochemistry, arterial blood gases, metabolic, cytogenetic, MLPA screening for chromosomal micro-aberrations and DNA analysis for the *FMR1* gene. Medical history of patients with epilepsy included information about the seizure characteristics, frequency, used antiepileptic drugs, and therapeutic responses on seizures and behavior. Additional investigations were administered to some of the patients based on their clinical and laboratory signs. 

Children under 5 years of age were tested with a developmental profile test to assess their neurodevelopment, and older children underwent intelligence tests (Stanford–Binet Intelligence Scale and Wechsler Intelligence Scale for Children) to establish their IQ. Intellectual disability (ID) was classified as mild (IQ 50–70), moderate (IQ 30–49), severe (IQ 20–29) or profound (IQ < 20).

All patients with an epileptic seizure underwent an EEG once or more (26 patients). An EEG was also performed on some of the children with ASD without seizures (seven patients). The types of epileptic seizures and syndromes were classified according to the ILAE 2017 Classification of seizure types by two epileptologists [25]. The frequency of epileptic seizures was estimated per day.

The EEGs were interpreted by two epileptologists and the abnormalities were classified as focal epileptiform, generalized epileptiform, hypsarrhythmia, slow wave activity, disorganized or slow background.

According to the etiology, patients were classified into three groups: symptomatic or syndromic (with proven etiology), probable syndromic (with suspicious but not proven etiology because additional investigations were not administered according to the parents’ wishes) and idiopathic (lacking clinical or laboratory evidence for a possible syndrome).

The patients were observed for 3–56 months.

### Statistical Methods

Parametric and non-parametric, correlative, linear and non-linear regressive analyses were applied using SPSS 23.0 (IBM Corp.,Armonk, NY, USA) software. Different tests of statistical significance were applied as appropriate, specifically the t-test, Fisher exact test, chi-square test, Kruskal–Wallis H test (*p* < 0.05 was considered statistically significant), as well as Spearman correlation coefficient.

This work was carried out in accordance with the Code of Ethics of the World Medical Association (Declaration of Helsinki) for experiments involving human subjects.

## 3. Results

**Figure 1:**
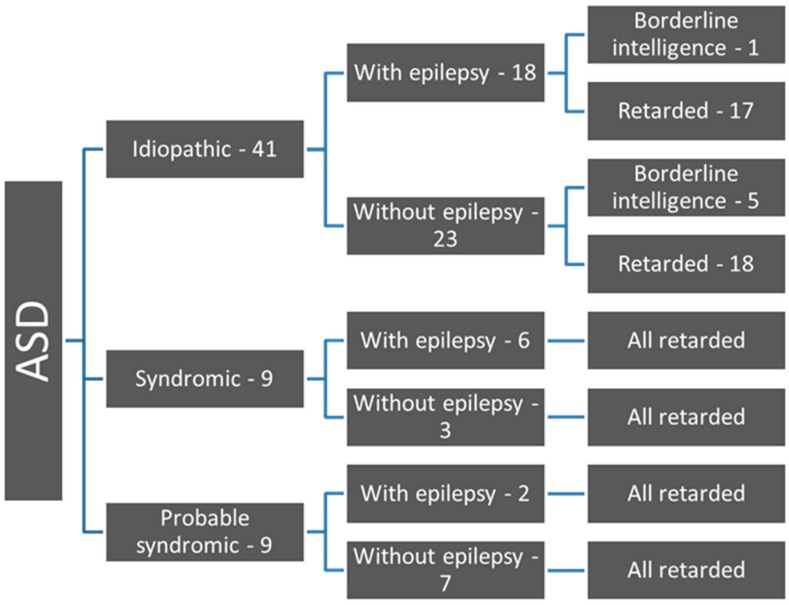
Flow chart of ASD patients according to etiology, epilepsy and ID.

The possible etiology of ASD in our patients is presented in Figure 1.

The etiologies of syndromic ASD patients were: chromosomal aberrations (four patients); Tuberous sclerosis complex (TSC) (two patients); Neurofibromatosis I (one patient); Rett syndrome (one patient); ESES (one patient). The suspected etiologies of probable syndromic ASD were: chromosomal micro-aberrations (three patients); mitochondrial disorders (two patients); monogenic (four patients, two of them with PTEN mutation).

**Figure 2:**
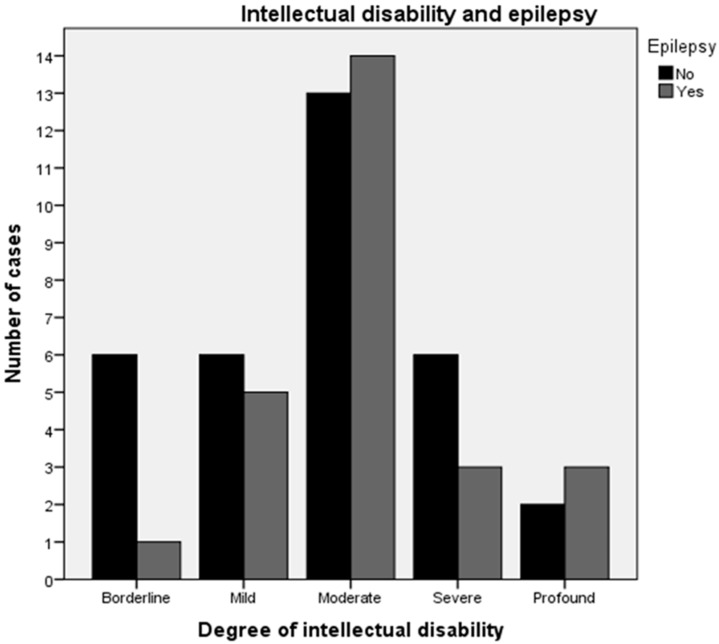
Epilepsy in different degrees of ID.

ID occurred as a comorbidity in 90% of our ASD patients (Figure 1). Borderline neurodevelopment or intelligence was established in 10% of ASD, only in idiopathic ASD. All symptomatic cases were mentally retarded (*p* > 0.05). The presence of epilepsy in ASD patients was 44.4% (26 out of 59 children). The occurrence of epilepsy in the three different etiologic groups is presented in Figure 1. Children with syndromic ASD tend to be more likely to have epilepsy (six out of nine children), but there was no significant difference (*p* > 0.05). The occurrence of epilepsy in different degrees of ID is presented in Figure 2.

**Figure 3:**
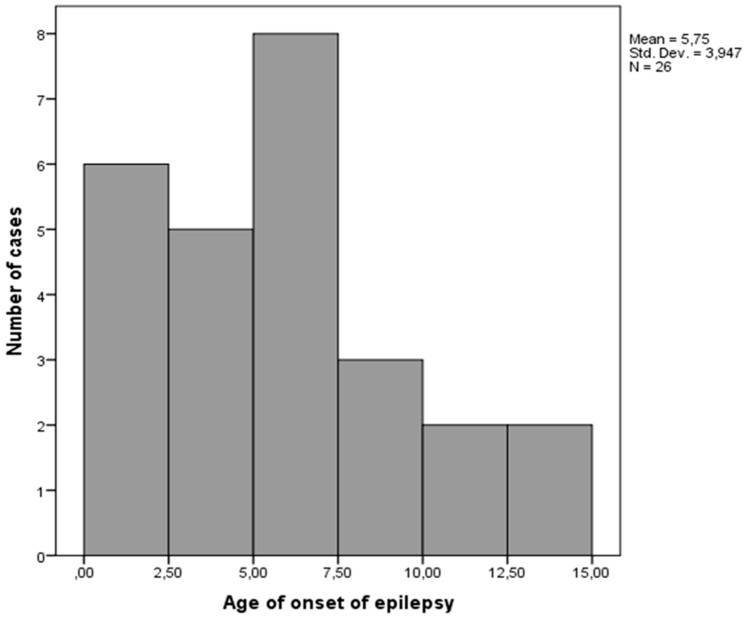
Age distribution of the onset of epilepsy (years).

The onset of epilepsy varied from 1 month to 14 years of age, but prevailed before 7 years of age. The onset was in the first year of life in four of 26 patients. The age distribution is presented in Figure 3.

The frequency of epileptic seizures was different (from a single seizure to numerous per day).

The types of epileptic seizures are presented in Table 1. All seizure types occurred in patients with ASD, but focal seizures were significantly more frequent (*p* < 0.001). Epileptic spasms preceded ASD in three of 26 patients (two of them with tuberous sclerosis complex, one with probable syndromic ASD).

Epileptic seizures preceded the diagnosis of ASD in eight of 26 patients.

In our study, three variables (IQ or developmental quotient, ID and degree of ID) were used as dependent variables, while the independent variables were the presence of epilepsy, age of onset of epilepsy, frequency of seizures, seizure type, sleep-EEG patterns, awake-EEG patterns and therapeutic response (Table 2 and Table 3). There was no correlation between epilepsy and either occurrence of intellectual disability or its degree. However, there was a correlation between the frequency of epileptic seizures and the degree of ID (more frequent seizures correlated with a more severe ID).

There was no significant relationship between gender and ASD etiology, ID or epilepsy. 

Focal epileptiform abnormalities prevailed on the EEGs in 14 of 26 children (*p* < 0.001). Generalized epileptiform activity was found in seven of 26 ASD children. One child had both epileptiform activities. One child had ESES.

In two of seven (28.6%) ASD patients without epileptic seizures, EEG showed focal epileptiform activity, but without ESES.

Epilepsy was treated with different antiepileptic drugs, according to the type of seizures and EEG changes, as monotherapy (20 patients) or polytherapy (six patients). The most commonly used drug was valproate (17 patients), followed by levetiracetam (six patients). Therapeutic response to seizures was good: 58% of the sample were seizure-free, and 27% had more than a 50% reduction of seizures. Therapeutic resistance was established in 15% of epileptic ASD patients, but no relationship (*p* > 0.05) was found between different ASD etiologies. There was no improvement in the autistic behavior after remission of epilepsy according to the parents’ opinion. 

## 4. Discussion

The comorbidity of epilepsy and ASD is well known in the literature and the occurrence of epilepsy in ASD ranges from 5–46% [2,3,4,5,6,7,8,9]. According to a recent population-based study of Jokiranta et al., the rate of epilepsy in ASD population was 6.6% [5]. Amiet’s meta-analysis of all articles from 1963–2006 pointed out that epilepsy occurred more commonly in syndromic autism, female autistic individuals, or ASD with ID (21.4% comparing to 8% without ID) [4]. The frequency of epilepsy in our ASD population was 44%. This relatively high percentage could be explained by the fact that this study was performed on hospitalized patients and by the high rate of co-occurring ID (90%). In contrast to our population, in Jokiranta et al. ID was present only in 12.7% of ASD patients [5]. We did not find any correlation between epilepsy and ID or the severity of ID. This could be due to the small proportion of non-mentally retarded ASD patients (10% with borderline intelligence). Most studies have found such a correlation; there was a higher percentage of epilepsy (46%) in ASD individuals with IQ < 40 according to Ameit’s meta-analysis [4]. However, in our study 70% of ASD patients had IQ < 50 and this could explain the high frequency of epilepsy (44%). 

Viscidi et al. found by multivariate logistic regression analysis independent associations between epilepsy and either older age or lower cognitive ability; other risk factors, such as poor language and developmental regression, were not associated with epilepsy after controlling for IQ [6]. We did not find correlation between epilepsy and ID or the degree of ID (*p* > 0.05).

Some studies found that the frequency of epilepsy was significantly higher in syndromic ASD compared to that in idiopathic ASD. The reason for this could be common neuropathological mechanisms in ASD, epilepsy and ID. We did not establish a significant difference, but ID prevailed in syndromic ASD. Many monogenic encephalopathies do not have any clinical features suspicious for syndromic ASD and some idiopathic ASD cases could really be monogenic encephalopathies. This could be a possible explanation for the lack of significant difference of the frequency of epilepsy among the different etiologic groups.

Bimodal age peaks of onset of epilepsy in ASD are mentioned in the literature to be early years of childhood and early puberty (above 10 years of age) [26]. According to our results, as well as those of Matsuo et al. and Jokiranta et al., the most frequent age of onset of seizures in ASD was during early childhood [5,27], while Hara showed increased risk of epilepsy in puberty [7]. Viscidi et al. also pointed out that children above 10 years of age are at increased risk for epilepsy (OR = 2.35) [6]. Epilepsy persists in adulthood in up to 80%, with remission in about 16%, while the remission rate in our sample was above 50% [8]. However, most of our patients were not followed beyond puberty.

Jokiranta et al. established that the number of children who had first received an ASD diagnosis prior to epilepsy and ID diagnosis was approximately the same as the number of cases first receiving an epilepsy diagnosis and later an ASD and ID diagnosis [5]. In our study epilepsy preceded the diagnosis of ASD in 30.7% of patients with ASD and epilepsy.

All types of seizures can occur in ASD individuals, but according to some studies complex partial seizures prevail (in three of four children with ASD and epilepsy) [28]. Our study confirmed these results, because more than half of the patients with ASD and epilepsy had focal seizures with or without secondary generalization. Six of seven children with autism and epilepsy in the study by Olsson et al. had more than one type of seizure and three of six had three or more types [28]. In our study only 15.4% of patients had more than two types of seizures. Sometimes there are difficulties in differentiating focal seizures such as complex partial seizures from stereotypic movements, so many videos and especially video-EEG could contribute to proper diagnosis.

Three of our 26 ASD patients with epilepsy had infantile spasms preceding ASD, two of them with TSC. 

Some of the epileptic encephalopathies could present as ASD. Srivastava et al. pointed out that 34 out of 62 genes of epileptic encephalopathies were also responsible for ASD and concluded that the gene defect could determine all the symptoms of ASD, epilepsy and ID [20]. Those genes can affect synapse formation, action potential generation, neurotransmitter release and excitatory inhibitory balance, thus leading to disrupted neuronal circles and networks [13,20].

Epileptic encephalopathies are characterized by epileptiform abnormalities associated with progressive cerebral dysfunction (cognitive and behavioral) and epileptic seizures, which are usually pharmacoresistant [29]. The etiology of epileptic encephalopathies could be genetic, metabolic or structural. ILAE identifies eight syndromes under epileptic encephalopathies: early myoclonic encephalopathy, Ohtahara syndrome, West syndrome, Dravet syndrome, myoclonic status in non-progressive encephalopathies, Lennox–Gastaut syndrome, LandauKleffner syndrome and epilepsy with continuous spike waves during slow wave sleep or ESES. However, in recent years more syndromes have been added to the spectrum of epileptic encephalopathies [30,31].

It might be speculated that epilepsy-like infantile spasms could determine ASD, because of the sure association between ASD and infantile spasms. The rate of ASD is higher not only after infantile spasms in TSC, but also in the general population with infantile spasms [32]. On the other side, in TSC, not all patients with infantile spasms develop ASD, and not all patients presenting with ASD follow infantile spasms. In our study three of 26 ASD patients with epilepsy had had infantile spasms, and only two of them had TSC. The incidence of ASD following infantile spasms is higher in more retarded children [33]. The authors pointed out that the etiologic factor determined the development of ASD after infantile spasms, and early treatment of infantile spasms did not prevent the development of ASD [33].

In our sample there were no patients with Dravet syndrome, despite the fact that the frequency of ASD in Dravet syndrome reaches up to 65% [34,35].

In our study one of 59 patients was diagnosed with ESES, but proper treatment and the disappearance of electrical status epilepticus occurred 3 years after the onset of the symptoms. This could be the reason for the persistence of the autistic features and ID. The hypothesis in ESES is that the seizures or the interictal epileptiform activity are responsible for the cognitive, language and behavioral deterioration [31]. Although epileptiform EEG abnormalities occur in 28% of children with autism and language regression, ESES is a rare occurrence in association with autistic regression [36]. The behavioral phenotypes of ESES and autistic regression overlap. The onset of ESES is usually between 4 and 8 years of age with global regression, while the regression of language in ASD occurs between 18 and 24 months, together with the appearance of autistic behavior. According to some authors, the differences in the age of regression, degree and type of regression, frequency of epilepsy and EEG abnormalities suggest that ESES and ASD are distinct phenotypes [24]. However, in some cases early onset ESES could be difficult to differentiate from ASD, so we suggest performing an EEG both awake and during sleep on all patients with autistic regression, especially when epileptic seizures co-occur. This could prevent the underdiagnosis of ESES, which is very important because its early treatment leads to a better prognosis.

EEG epileptiform abnormalities were found in 35% to 86% of ASD individuals with epilepsy, and up to 60% of ASD individuals without epilepsy, according to the literature [4]. According to Ballaban-Gil‘s study, epileptiform discharges (EDs) are often more common in children with autistic regression, even in the absence of a history of epilepsy [37]. We found epileptiform discharges in 84.6% of ASD patients with epilepsy and in 28.6% (two of seven) of those without epilepsy, but these two patients without epileptic seizures had no ESES. One of 59 patients had ESES and his behavior and cognition improved, but did not normalize after the disappearance of this EEG phenomenon. It is still a disputable question whether EEG epileptiform abnormalities without epileptic seizures should be treated, and there is not a simple answer [38,39,40]. In our study there was no improvement in autistic symptoms after epilepsy remission, even after the disappearance of the epileptiform discharges. We can speculate that epileptiform discharges could be due to an underlying etiological reason, probably encephalopathy resulting in ASD and ID as well. Sanchez Fernandez et al. concluded that in patients with epileptic encephalopathies and cognitive dysfunction/regression, which can be related to epileptiform discharges, a treatment trial might be justified [41].

There are evidences in the literature that in the short term interictal epileptiform discharges could disrupt neuronal processes and cognition [8,39]. However, there are few non-randomized controlled studies with evidence of the positive effect of the disappearance of these discharges on cognitive functions and behavior [40,41]. At present, there is no strong evidence for or against the treatment of asymptomatic epileptiform discharges. Further studies are needed to conclude if it is possible to improve the autistic symptoms by treating the EEG interictal epileptiform discharges. However, wehighly recommend sleep-EEG to all ASD patients with epileptiform discharges. This will prevent the underdiagnosis of ESES, and can improve autistic symptoms and cognition if treated in time.

The prevalence of ASD among epileptic patients is also higher than in the general population, so the common neurobiological bases could be the explanation for this comorbidity, often associated with ID [27,42,43]. Bozzi et al. concluded that an excitation/inhibition balance resulting from neurodevelopmental deficits of multiple origins, including genetic causes, might represent a common pathogenic mechanism for both ASD and epilepsy by reviewing the most significant studies supporting this hypothesis [13].

Usually the treatment of epilepsy in ASD is based on the general principles of treatment of epileptic seizures with traditional antiepileptic drugs (AEDs). Several lines of evidence point to valproate, lamotrigine and levetiracetam as the most effective and tolerable AEDs for individuals with ASD [44]. We also used valproate, followed by levetiracetam with good therapeutic response to seizures (remission), but without improvement of autistic behavior.

Regarding the opinion that the gene defect could determine all the symptoms, new therapeutic options were suggested for ASD, epilepsy and ID based on the gene defect disturbance, including GABA agonists, modulators of GABA A receptors, modulators of GABA metabolism, glutamate receptor antagonists, insulin-like growth factor 1 and m-TOR inhibitors for ASD–epilepsy comorbidity. (13,19). M-TOR inhibitors had hopeful results in patients with TSC, ASD and PTEN-related disorders. A paradoxical response to treatment with conventional GABAergic agonists has been reported in ASD [44,45,46,47,48,49,50], as well as a lower effectiveness of benzodiazepines in certain forms of ASD–epilepsy such as 15q11.2 duplication [19].

## 5. Conclusions

Our study showed a significant percentage of epilepsy among an ASD population and more than one fourth were of probable symptomatic etiology. Those could be managed with specific treatments based on the pathophysiology of the gene defect. Intellectual disability co-occurred in nearly all cases, and borderline neurodevelopment or intelligence occurred only in idiopathic ASD. There was no significant difference of epilepsy rates in different etiologic, gender and ID groups, probably because of the high percentage of ID and because this was a hospital-based study. Further evidence-based research on comorbid epilepsy–ASD–ID cases are needed to approve these possible novel therapeutic strategies for ASD with known etiologies and gene dysfunctions.

## Figures and Tables

**Table 1:** Type of seizures in patients with ASD and epilepsy.

Type of Seizures	*N* = 26	%
Focal with or without secondary generalization	14	53.4
Generalized tonic-clonic	5	19.2
Absences	2	7.7
Polymorphic seizures	4	15.4
Preceding infantile spasms	3	11.5

**Table 2:** Correlative analysis of different variables: epilepsy, age of onset of epilepsy, frequency of seizures, seizure type, sleep-EEG patterns, awake-EEG patterns and therapeutic response.

Relationship	Chi-Square	d*f*	*p*-Value
ID*Epilepsy	2.035	1	0.154
ID*Type of seizures	4.167	4	0.384
ID*Sleep-EEG	2.513	4	0.642
ID*Age of onset of epilepsy	0.264	1	0.607
ID*Frequency of seizures	2.189	1	0.139
ID*Awake-EEG	2.640	4	0.620
ID*Therapeutic response	0.124	1	0.725
ID degree*Epilepsy	3.200	3	0.362
ID degree*Type of seizures	10.069	12	0.610
ID degree*Age of onset of epilepsy	0.582	3	0.901
ID degree*Frequency of seizures	4.391	3	0.222
ID degree*Sleep-EEG	8.370	12	0.756
MR degree*Awake-EEG	11.467	12	0.489
ID degree*Therapeutic response	3.427	3	0.330
Intelligence*Epilepsy	0.198	1	0.656
Intelligence*Type of seizures	1.688	4	0.793
Intelligence*Sleep-EEG	1.328	4	0.857
Intelligence*Awake-EEG	5.137	4	0.274
Intelligence*Therapeutic response	0.116	1	0.733

**Table 3:** Correlative analysis of different variables: intelligence; age of onset; frequency of seizures.

Correlation	Spearman’s Rho	*p*-Value
Intelligence*Age of onset of seizures	0.180	0.368
Intelligence*Frequency of seizures	−0.505 *	0.033
